# The Feasibility of Developing a Universal SARS-CoV-2 Vaccine

**DOI:** 10.3390/vaccines14030259

**Published:** 2026-03-13

**Authors:** Mohammed Asaad, Mohamed O. Mustafa, Yaman Al-Haneedi, Lina Shalaby, Rania shams Eldin, Yasar Mohamedahmed, Hadi M. Yassine, Abdallah M. Abdallah, Mohamed M. Emara

**Affiliations:** 1 Basic Medical Sciences Department, College of Medicine, QU Health, Qatar University, Doha 2713, Qatar; mohammedasaad@qu.edu.qa (M.A.); momer.ahmed@qu.edu.qa (M.O.M.); rania.aly@qu.edu.qa (R.s.E.); abdallah.musa@qu.edu.qa (A.M.A.); 2 School of Medicine, Wayne State University, Detroit, MI 48201, USA; 3 Hamad Medical Corporation, Doha 3050, Qatar; v-ymohamedahmed@hamad.qa; 4 Biomedical Research Centre, QU Health, Qatar University, Doha 2713, Qatar; hyassine@qu.edu.qa

**Keywords:** SARS-CoV-2 variants, SARS-CoV-2 universal vaccines, new vaccine platforms, spike protein, conserved region

## Abstract

As SARS-CoV-2 continues to evolve with increased transmissibility and immune evasion, the need for vaccines that provide broader and more durable protection has become increasingly urgent. The extensive research spurred by the pandemic has accelerated the development of diverse vaccine platforms, including mRNA, DNA, virus-like particles (VLPs), recombinant proteins, and mosaic mono- and polyvalent vaccines. While several of these platforms have reached regulatory approval and widespread clinical employment, others remain under evaluation or in various stages of clinical development. These vaccines have significantly reduced infection rates, severe disease, and hospitalizations, particularly among high-risk group. Nevertheless, the ongoing emergence of novel variants and subvariants has challenged the efficacy of both existing and newly developed vaccines. This evolving landscape underscores the urgent need for a universal SARS-CoV-2 vaccine platform capable of providing comprehensive and long-lasting immunity. In this review, we evaluate current and emerging strategies for SARS-CoV-2 universal vaccine development, with a focus on antigen design, breadth of immune protection, and clinical feasibility. Attention is given to various universal vaccine platforms such as the mosaic polyvalent spike construct, multi-epitope vaccines targeting the receptor-binding domain (RBD), and approaches centered on the conserved S2 subunit of the spike protein. We also discuss strategies leveraging additional conserved viral proteins and T helper (Th) and cytotoxic T lymphocyte (CTL) epitopes from across coronaviruses. By highlighting the advances in these areas, this review provides a framework to guide the rational design of next-generation universal vaccines capable of delivering broad and durable protection against SARS-CoV-2 variants.

## 1. Introduction

Since its emergence in December 2019, SARS-CoV-2 has continued to pose a major global public health threat. As of 25 June 2025, COVID-19 has caused over 675.5 million reported cases and more than seven million deaths worldwide [1]. Although widespread vaccine deployment substantially reduced morbidity and mortality, the virus has continued to spread, resulting in approximately 704 million reported infections to date [1]. In addition, the emergence of new SARS-CoV-2 variants and subvariants, such as subvariants LF.7.2.1, NP.1, and LP.8.1, has posed additional challenges. Furthermore, mutations in the receptor-binding domain (RBD) and N-terminal domain (NTD) have enhanced viral adaptability and transmissibility while increasing resistance to neutralizing antibodies elicited by existing vaccines [2]. Beyond rapid viral spread, COVID-19 inflicted profound economic damage worldwide [3]. The FDA fully approved the first COVID-19 vaccine on 23 August 2021, following EMA authorization, enabling global emergency deployment to curb transmission [4,5]. Since then, substantial progress has been made in developing a wide range of SARS-CoV-2 vaccines, which are currently in use or still in the pipeline. The huge advances in sequencing technologies and immuno-informatics tools have been utilized ideally in predicting and designing the epitopes of SARS-CoV-2, leading to the development of these numerous vaccines. According to the World Health Organization’s COVID-19 Vaccine Tracker (last updated 2 December 2022), 821 vaccine trials were underway across 80 countries. Of these, 242 vaccine candidates were in various stages of development: 66 in phases 1, 72 in phase 2, and 92 in phase 3. Despite this extensive global pipeline, only 50 vaccines had received regulatory approval [6]. This remarkably large number of vaccine trials reflects the substantial worldwide effort invested in controlling the pandemic; yet, despite these efforts, viral transmission has continued. Collectively, this unprecedented pandemic underscores the urgent need for next-generation vaccines capable of providing broad and durable protection against both current and emerging SARS-CoV-2 variants. One promising strategy toward achieving this goal is the development of a universal SARS-CoV-2 vaccine, a concept whose feasibility and scientific foundations will be discussed in this review.

## 2. SARS-CoV-2 Evolution and Immune Escape Mechanisms

The rapid evolutionary rate of SARS-CoV-2 has been driven by the accumulation of numerous mutations in the spike protein and other structural proteins, including the nucleocapsid [7,8]. Mutations in the SARS-CoV-2 spike protein are responsible for two key changes: stronger binding to the ACE2 receptor and impaired antibody cross-neutralization efficacy [9]. The substitutions within the Omicron variants account for nearly 9% of the total number of spike protein residues of the ancestral Wuhan strain [10]. Furthermore, both pre-Omicron and post-Omicron variants possess distinct structural features that have optimized viral fitness and shaped the evolutionary trajectories of subsequent emerging subvariants [11]. SARS-CoV-2 evolves rapidly due to high mutation rates, especially in the spike protein’s receptor-binding and N-terminal domains, as well as structural (e.g., nucleocapsid) and nonstructural genes. This is driven by selective pressure from population immunity via infection and vaccination [12], prolonged infection and superiority of the endogenous IgM immune response in an immunocompromised patient [13], and transmission bottlenecks favoring enhanced ACE2 binding (e.g., Omicron BA.1 S477N/Q493R/Q498R) [14,15]. The last evolution of SARS-CoV-2, marked by the transition from XBB to the BA.2.86/JN.1 lineage combined with waning of antibody response against XBB to JN.1, underscores the necessity of JN.1-based vaccine boosters, particularly those targeting the emerging KP.2 and KP.3 sub-lineages [16,17]. Phylogenetic analyses reveal consistent evolutionary rates and seasonal variant emergence [18], necessitating careful vaccine antigen selection and updated boosters. Antigenic cartography reveals that although Omicron BA.1 and BA.2 form clusters distinct from pre-Omicron variants, later lineages such as BA.4/5, BQ, XBB, and JN.1 show even greater antigenic divergence, further widening their topographical distance from ancestral strains [19,20]. Moreover, a significant portion of the recurrence and enhanced fitness of later Omicron lineages can be attributed to five key substitutions (R346, K444, L452, N460, and F486) [21], which collectively underscore their evolutionary role in facilitating immune escape. The analysis of the B-cell receptor (BCR) repertoire during the XBB-to-JN.1 transition reveals potent activation of memory B cells producing cross-reactive neutralizing antibodies (NAbs). However, KP.2 and KP.3 variants escape neutralization even from JN.1-induced NAbs [16], while recent strains (LF.7.9, XEC.25.1, XFH, XFG) show strong escape via A475V/N487D shifts; AB.3.2 resists antibodies but lacks ACE2 affinity/infectivity for dominance [22]. Convergent RBD mutations, partly driven by immune imprinting, drive much of the observed immune escape while preserving ACE2 affinity and viral fitness [17,23]. Spike RBD substitutions enhance antibody evasion but may reduce ACE2 binding and infectivity, necessitating careful vaccine antigen updates to balance escape and fitness. Consequently, updating vaccine antigens demands careful evaluation to balance immune escape with the preservation of viral fitness. Taken together, these findings illustrate a dynamic and relentless evolutionary arms race between SARS-CoV-2 and the human immune system. The data clearly shows that immune pressure from both infection and vaccination acts as the primary catalyst for the emergence of variants with enhanced fitness and immune-evasive properties. The consistent pattern of antigenic divergence from pre-Omicron strains to the highly distinct XBB and JN.1 lineages, and now to KP.2/KP.3, demonstrates that the virus is not evolving randomly but through convergent evolution on key spike protein residues that balance immune escape with preserved ACE2 binding affinity. This cycle underscores a fundamental challenge: our current vaccine strategies remain reactive, constantly chasing the latest antigenic shift rather than preempting it, highlighting the urgent need for next-generation approaches that can induce broader and more durable immunity.

## 3. Current Strategies for SARS-CoV-2 Vaccine Platforms

In response to the SARS-CoV-2 pandemic, scientists rapidly developed a diverse array of vaccine platforms under emergency use authorizations (EUAs) to combat the virus. These platforms can be broadly categorized into two main groups: whole virus vaccines and component viral vaccines. By December 2022, approximately 50 COVID-19 vaccine candidates had received regulatory approval, encompassing a wide range of platforms: non-replicating viral vector vaccines, 11 inactivated vaccines, 19 protein subunit vaccines, several virus-like particle (VLP) vaccines, nine RNA vaccines, and one DNA vaccine [6]. Although inactivated COVID-19 vaccines played a crucial role in China’s initial vaccination campaign, they face notable challenges in development, manufacturing, safety, and efficacy across certain populations [24]. Conversely, component viral vaccines utilize specific viral elements, including protein subunits, nucleic acid-based approaches (such as DNA and RNA), and virus-like particles (VLPs), and have demonstrated remarkable success in terms of safety, scalability, and adaptability [25,26]. Advances in mRNA technology and immuno-informatics significantly accelerated the use of mRNA as both a therapeutic agent and vaccine platform [27], offering rapid design, scalable production, and easy administration [28], making them highly promising candidates for a wide range of pathogens. There are two main types of mRNA vaccines: self-amplifying and non-replicating (modified and unmodified) [29,30,31]. Both can induce robust neutralizing antibody responses, a critical component of protective immunity [27,31]. Self-amplifying RNA (saRNA) vaccines encode both target antigens and viral enzymes that play a key role in viral replication, including the SARS-CoV-2 spike protein and a viral replicase, respectively. This ability to induce immune responses against more than one target simultaneously has enabled the use of lower doses, thereby increasing vaccine efficiency and reducing production costs, making them a promising platform for large-scale deployment. Non-replicating mRNA vaccines, whether modified for stability or unmodified, have simpler designs and proven efficacy in approved COVID-19 vaccines. Ongoing research focuses on balancing complexity, scalability, and immune potency to optimize both platforms for broader use, including emerging infections and cancer immunotherapy. Non-replicating mRNA vaccines, such as Pfizer-BioNTech and Moderna, demonstrated ~93–94% efficacy against symptomatic COVID-19 and showed the highest total efficacy and effectiveness compared to other available vaccines [32]. Despite their high efficacy, they require booster doses to maintain adequate immunity, particularly against newly emerging variants [33]. A third type of RNA vaccines, circular RNA (circRNA), has also emerged as a promising platform [34]. CircRNA vaccines have greater stability and thus demonstrate prolonged half-lives compared to conventional linear mRNA vaccines [35,36]. Circular RNA vaccines, with their prolonged stability and sustained antigen expression, can elicit durable, high-quality immune responses and may overcome key limitations of current mRNA vaccines, enabling broader protection against diverse SARS-CoV-2 variants. While most SARS-CoV-2 vaccine candidates focus on the spike protein because of its strong immunogenicity, circRNA platforms could also facilitate targeting additional conserved viral antigens to enhance breadth of protection [37]. Recent advances in genome-modified live-attenuated vaccines (LAVs) have demonstrated durable protection against both wild-type and Omicron variants, with no detectable transmission [38]. Virus-like particle (VLP) vaccines also represent a promising strategy, as they closely mimic native viral structure and can elicit potent, long-lasting immune responses, offering clear advantages for next-generation and potential universal vaccine designs. In addition, a mosaic homotypic nanoparticle vaccine incorporating eight receptor-binding domains (RBDs) of SARS-CoV-2 and animal beta coronaviruses elicited protective immune responses against zoonotic coronaviruses [25,39]. Collectively, a wide range of vaccine strategies have been developed, each with distinct advantages and disadvantages in combating COVID-19. Careful selection, adoption and effective implementation of those strategies is critical for driving the development of next-generation COVID-19 vaccine platforms. Indeed, the rapid development of these diverse vaccine platforms reveals a critical discussion about the trade-offs between speed, efficacy, durability, and breadth. The initial pandemic response prioritized speed and immediate efficacy, which was controlled by non-replicating mRNA vaccines. However, their reliance on frequent, variant-specific boosters has exposed the limitations of a purely reactive strategy. The subsequent innovations outlined here can be understood as a strategic branching point. On one branch, platforms like saRNA and circRNA represent an optimization of the current mRNA model, seeking to improve its cost, stability, and the longevity of the immune response. On a more ambitious branch, strategies employing VLPs, genome-modified LAVs, and particularly mosaic nanoparticles signal a fundamental shift in ambition: moving away from chasing individual variants and toward inducing broad, cross-reactive immunity against entire sub-genera of coronaviruses.

**Figure 1:**
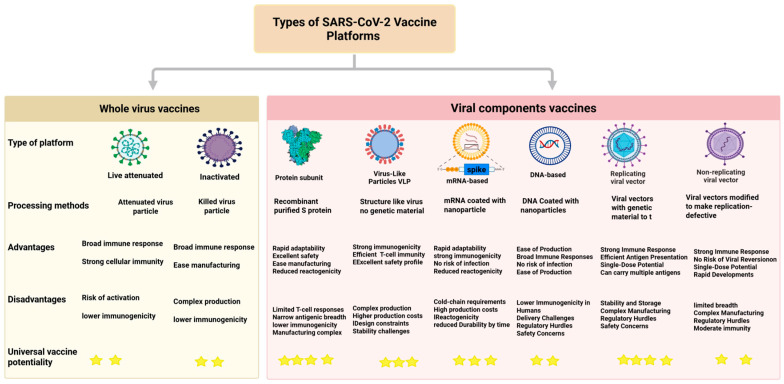
Comparative overview of major vaccine platforms, highlighting their advantages, disadvantages, and potential for universal vaccine development. Platforms include live attenuated, inactivated, protein subunit, virus-like particles, and DNA-based vaccines. Universal vaccine potential is indicated by star ratings.

Figure 1 illustrates the current vaccine platforms employed during and after the pandemic. These approaches will support the rapid development of novel vaccines that can counter emerging variants and reduce future zoonotic spillover, while prioritizing platforms with the greatest potential for universal vaccine protection.

## 4. Effectiveness of Current SARS-CoV-2 Vaccines and Variant Evasion

On 6 April 2024, the World Health Organization (WHO) reported significant genetic and antigenic evolution of the SARS-CoV-2 spike protein, reinforcing the necessity for ongoing genomic surveillance and dynamism of vaccine strategies to address emerging variants [40]. A substantial portion of the global population has been vaccinated with approved SARS-CoV-2 vaccines, which primarily target the receptor-binding domain (RBD) of the spike protein. However, numerous mutations have been identified in RBD variants of interest (VOCs) [41]. Early COVID-19 vaccines demonstrated strong efficacy against initial variants; for example, AstraZeneca showed 70% effectiveness against symptomatic Alpha infections, while Pfizer achieved ~90% efficacy [42]. Omicron’s emergence markedly reduced vaccine effectiveness, with studies reporting declines to around 50% [43,44]. Despite maintaining strong protection against severe disease and mortality, these vaccines provided limited defense against mild infection [44]. In Qatar, the BNT162b2 vaccine demonstrated 95.9% effectiveness (95% CI: 94.0–97.1) against severe and critical cases following the primary series of vaccination [45]. However, protection waned rapidly six months after the second dose [46]. In Belgium, primary vaccination transmission efficacy was 96% (Alpha), 87% (Delta), and 31% (Omicron), declining further to 71% (Delta) and 55% (Omicron), with similar trends in Qatar [47]. An evaluation of England’s three main COVID-19 vaccines Oxford/AstraZeneca (AZD1222), Pfizer-BioNTech (BNT162b2), and Moderna (mRNA-1273) showed sustained protection against hospitalization and death for ~3 months, followed by gradual decline to moderate levels after one year [48]. Furthermore, primary vaccine series offered limited efficacy against Omicron and subvariants due to substantial immune escape from both vaccine- and infection-induced immunity [47,49], including evasion of humoral and cell-mediated neutralization [50,51]. Notably, Omicron subvariants such as XBB.1.5, CH.1.1, and CA.3.1 demonstrated complete resistance to y antibodies induced by Wuhan-based antigens, even after a third dose and bivalent booster [52,53]. This immune escape, driven by spike protein mutation that enhances fusogenicity [53], significantly undermined primary vaccine series, dropping efficacy against infection from >80% (early strains) to <40% due to mutations disrupting key epitopes in both humoral (antibody) and cell-mediated (T-cell) arms. While hybrid immunity from prior infection offered partial restoration, widespread immune evasion, prolonged transmission and frequent breakthrough infections underscored the need for boosters, variant-adapted antigens, or conserved-target platforms to regain comprehensive protection [54]. Emerging variants and subvariants have further reduced neutralizing antibody responses.

Altogether, this evidence redefines our understanding of vaccine “effectiveness” in the context of a rapidly evolving respiratory virus. The data clearly shows a strategic shift in the role of first-generation vaccines: from preventing infection and transmission against early variants to primarily mitigating severe disease and death against Omicron and its sub-lineages. This is not a story of vaccine failure, but rather a compelling, real-world demonstration of antigenic drift, where mutations in key RBD epitopes systematically eroded the neutralizing capacity of both humoral and cellular immunity induced by the ancestral spike protein. The significant contrast in efficacy figures before and after Omicron’s emergence serves as a crucial lesson, illustrating the inherent limitations of a static vaccine design against a highly plastic pathogen and creating an undeniable mandate for the development of variant-adapted boosters and next-generation, broader-spectrum vaccine platforms.

Table 1 summarizes current data on neutralization reduction and underlying mechanisms for available vaccines.

## 5. Current COVID-19 Vaccine Challenges

Despite the success of approved vaccines in reducing SARS-CoV-2 transmission and mitigating the COVID-19 pandemic, significant challenges persist. mRNA vaccines proved highly effective and enabled rapid development, yet their stability continues to pose challenges for widespread distribution and accessibility. Additionally, the ongoing emergence and re-emergence of COVID-19 variants continue to threaten global health, placing considerable strain on healthcare systems and economies [59]. The short duration of the vaccine effectiveness, adverse effects, and challenges in achieving herd immunity have reduced overall coverage and efficacy [60,61,62]. Recent reports from 4 November 2024 indicate a resurgence of COVID-19 transmission across multiple countries, with confirmed cases exceeding 19,000 in the last month [63]. The continued emergence of new variants despite existing vaccines poses a major challenge to pandemic control and vaccination efforts [64]. The rapid evolution of SARS-CoV-2 from the prototype to Beta, Gamma, Delta, and Omicron variants has compromised vaccines’ effectiveness and led to breakthrough infections [65,66], highlighting the urgent need for booster doses that elicit cross-protective T-cells immunity [67]. Consequently, the durability of protection offered by current mRNA vaccines remains limited [68]. Together, these challenges highlight the need for a universal vaccine offering broad protection against evolving SARS-CoV-2 variants.

## 6. Optimizing an Antigen Selection for Universal SARS-CoV-2 Vaccine

The high mutation rates of SARS-CoV-2 underscore the urgent need to design effective universal vaccines capable of addressing current variants of concern and future emerging strains. Identifying and engineering potent immunogenic targets through innovative approaches is critical for developing universal COVID-19 vaccine platforms that provide broad protection against both existing and evolving viral variants. These approaches should focus on developing vaccines that provide broad protection against emerging variants and cross-protection against zoonotic coronaviruses [69]. Most of the current vaccines predominantly target the spike protein as the principal protective antigen, due to its critical role in viral entry [70,71]. An ideal approach to developing universal SARS-CoV-2 vaccine is to enhance protection and efficacy offered by already existing mRNA-based vaccines. A promising strategy through which this can be achieved is codon de-optimization, a technique previously applied to several viruses [72,73], resulting in viral attenuation, enhanced immunogenicity, and protection against challenge with parental strains. By optimizing mRNA sequence to increase stability, it can further improve vaccine efficacy and durability, while potentially boosting immunogenicity and broad reactivity, ultimately leading to robust neutralizing antibody responses against variants of concern [74,75]. Divergent mutations in the RBD and NTD domains of the spike protein, which are key targets for neutralizing antibodies, have driven the emergence of new variants [76]. Decoding these mutations offers a promising strategy for stabilizing vaccine antigens and enhancing their protective efficacy, potentially serving as a pivotal approach toward developing a universal vaccine. Moreover, extending stabilization to include the spike S2 subunits from SARS-CoV-2 as well as sarbecovirus such as SARS-CoV-1 (clade 1a) and PRD-0038 (clade 3) has conferred protection in mice against XBB.1.5 challenge [77]. Advanced computational tools are accelerating universal vaccine development. Machine learning (ML) and artificial intelligence (AI) will play a central role in this effort. AI pipelines are crucial for identifying conserved proteins and immunogenic T-cell epitopes across coronaviruses to enable cross-reactive immunity. Predictive models like EVE-Vax anticipate viral mutations, facilitating the design of vaccine antigens that strengthen defenses against emerging variants; integration of viral genomic structure with epidemiological data helps in forecasting high-risk mutations before they become widespread [78,79], which shifts our interaction with the virus pandemic from reactive responses to proactive pandemic prevention. In 2020, the Vaxign ML platform identified six proteins including the spike (S) protein and five non-structural proteins as highly protective antigens [80]; this strategy underscores the potential integration of this technology for leveraging antigens with broad antigenicity to develop universal vaccines while providing wide-spectrum protection against sarbecoviruses in general. Collectively, integrating optimized antigen design, conserved-target strategies, and AI-driven predictive tools offers a promising pathway toward universal SARS-CoV-2 vaccines capable of providing broad, durable protection against current and future coronavirus threats.

## 7. The Potential of Universal SARS-CoV-2 Vaccine

Developing a universal SARS-CoV-2 vaccine is vital for long-term disease control, offering broad protection against current and future variants while reducing the need for frequent boosters. The persistent evolution of SARS-CoV-2 and related coronaviruses constitutes a critical barrier to universal vaccine development. However, multi-subunit vaccines incorporating conserved spike RBD epitopes have demonstrated the capacity to elicit broadly neutralizing antibodies against multiple SARS-CoV-2 strains, including ancestral variants [81]. Furthermore, nanoparticle-based polyvalent protein cocktails exhibit potential for cross-protection against SARS-CoV-2-like viruses’ clade 1 sarbecoviruses [82] Nanoparticles displaying a mosaic of SARS-CoV-2 and animal betacoronavirus RBDs elicited antibodies with enhanced cross-reactivity against diverse RBDs, effectively neutralizing heterologous pseudotyped coronaviruses [39,83]. Additionally, *Helicobacter pylori*-assembled conserved epitopes from prior SARS-CoV-2 variants elicited durable and robust humoral and cellular immune responses [84]. As a result, leveraging multi-subunit vaccines targeting conserved RBDs and mosaic nanoparticle formulation represents a promising approach to broad-spectrum immunity and universal SARS-CoV-2 vaccine development, with potential cover emerging and re-emerging SARS-CoV-2 variants and related coronavirus.

Furthermore, the discovery of broadly cross-reactive monoclonal antibodies against animal sarbecoviruses, SARS-CoV-1, and SARS-CoV-2 in plasma of COVID-19 convalescents and vaccine recipients underscores a strong foundation for universal vaccine development [85]. Combining ancestral SARS-CoV-2 antigens with broadly neutralizing anti-sarbecovirus antibodies offers a promising strategy for universal vaccine design to prevent future SARS-CoV-2 and related sarbecovirus outbreaks.

This exploration of universal vaccine candidates moves the anticipation from chasing variants to a proactive strategy of anticipating viral evolution. On one hand, strategies like multi-subunit and mosaic-nanoparticle vaccines are essentially “teaching” the immune system to recognize the common architectural features shared by different coronaviruses. On the other, the identification of broadly neutralizing antibodies in convalescent individuals acts as a vital proof-of-concept, demonstrating that a universal response is not merely a theoretical construct but a tangible biological outcome that can be elicited and engineered. Therefore, we can predict a paradigm shift in vaccinology, where the strategic goal is no longer just to end the current pandemic but to establish a foundational, cross-protective immunity against an entire class of viral threats.

## 8. The Prosperity of SARS-CoV-2 S2 Subunits as a Universal Vaccine

The S2 subunit is highly conserved across SARS-CoV-2 variants and subvariants, as well as other betacoronaviruses, making it a promising universal vaccine antigen for broad protection against emerging variants. Broadly neutralizing antibodies (bnAbs) targeting the S2 subunit, isolated from vaccinated donors, confer protection against the three major betacoronaviruses SARS-CoV-1, SARS-CoV-2, and MERS-CoV that have emerged over the past two decades [86]. Notably, one bnAb epitope was highly conserved across most variants and retained conformational stability in both the pre- and post-fusion states of the spike protein [87]. These findings highlight the S2 subunit as a key target for universal coronavirus vaccine development. Immunization of two mouse models with a prefusion-stabilized SARS-CoV-2 S2 subunit, achieved via interprotomer disulfide bonds, elicited broadly neutralizing responses against multiple sarbecoviruses and protected against lethal challenge [88]. Moreover, combining two conserved sarbecovirus proteins, the S2 subunit and the nucleocapsid (N) protein successfully elicited broad systemic neutralizing antibody responses and robust Th1 immunity, as evidenced by IFN-γ secretion [89]. This synergy reveals the potential of the S2 subunit as a central component of universal coronavirus vaccine strategy and development. Owing to its high sequence conservation, structural stability across pre- and post-fusion states, and ability to induce broadly neutralizing and cellular immune responses, the S2 subunit represents a promising antigen for achieving durable, cross-protective immunity against current and emerging coronaviruses. Therefore, the focus on the S2 subunit represents a strategic move in SARS-CoV-2 vaccine design, deliberately moving away from the hyper-variable S1/RBD region that drives immune escape.

## 9. Identification of Non-Spike Protein Antigens as Universal SARS-CoV-2 Vaccine Candidates

**Figure 2:**
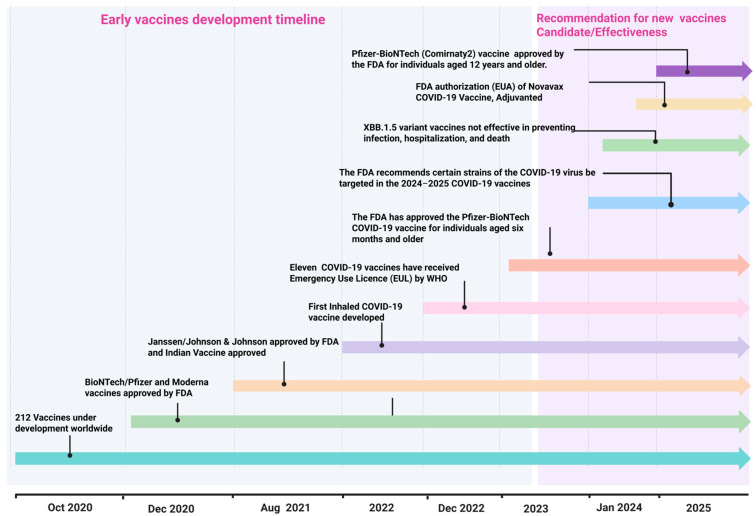
A chronological diagram of SARS-CoV-2 vaccine challenges. The timeline highlights key milestones in the design, approval, and evolution of vaccine platforms against SARS-CoV-2 variants. It shows both early approved vaccines and more recent candidates currently in development.

Another promising approach is targeting conserved non-spike antigens, as studies have suggested these highly conserved SARS-CoV-2 proteins can confer robust, broad cross-protective immunity against multiple variants of concern (VOCs) [90,91,92,93]. Ten conserved antigens have been identified, seven of which are preferentially recognized by both CD4^+^ and CD8^+^ T cells in unimmunized patients [90]. Furthermore, the combining of highly conserved antigens that target both B-cell and T-cell epitopes alongside with the Spike protein elicited cross-protective immunity, accelerated viral clearance, and mitigated lung pathology from highly pathogenic variants [90,91]. Moreover, a multi-epitope vaccine incorporating S1-RBD-sFc and conserved Th and CTL peptides from Sarbecovirus N, M, and S2 proteins induced a durable Th1-biased response characterized by IFN-γ production and robust cytotoxic CD8+ T-cell activity [92]. Conserved antigens from other proteins, such as the SARS-CoV-2 nucleocapsid (N) protein, a key factor in viral replication and pathogenesis [94], may serve as an optimal target for this approach, offering broader functionality [95]. Inclusion of the nucleocapsid (N) protein in vaccine design broadens the immune response by eliciting both humoral and cellular immunity, potentially providing longer-lasting protection against SARS-CoV-2 variants in hamsters [96,97]. Moreover, antibodies against nucleocapsid proteins have been detected in both unvaccinated and vaccinated individuals [98,99]. Additionally, the use of a highly conserved SARS-CoV-2 ORF3a ectodomain can elicit robust systemic anti-ORF3a antibody response and induced T-cell immune response in the lung [100]. Figure 2 illustrate the anticipated current and future directions of SARS-CoV-2 universal vaccine platforms. Although early studies suggested conserved proteins have low immunogenicity, the nucleocapsid (N), ORF3a ectodomain protein, and conserved domains within the spike protein remain strong candidates for next-generation universal COVID-19 vaccines, with the potential to induce both cellular and humoral immune responses.

## 10. Identifying Novel Spike Protein Antigens for Universal SARS-CoV-2 Vaccine Development

Although current vaccines have been highly effective in mitigating severe SARS-CoV-2 infections and mortality, the emergence of variants of concern (VOCs) and variants of interest (VOIs), coupled with their immune escape capabilities, underscores the need for ongoing development of enhanced vaccine strategies. One of these strategies involves identifying novel antigenic epitopes that can confer a strong and broad immune response. Utilizing a multivalent approach that incorporates multiple antigenic epitopes has shown promise in discovering new targets. For example, in BALB/c mice, a multivalent mRNA vaccine elicits broad neutralizing antibody responses against SARS-CoV-2 VOCs [101]. The high degree of conservation and structural stability positions of the S2 subunit marks a strategic starting point for identifying novel spike antigenic epitopes. S2-specific monoclonal antibodies from recovered human individuals bind conserved epitopes across multiple S2 regions and broadly neutralize SARS-CoV-2 variants, SARS-CoV-1 and related zoonotic sarbecoviruses [102], as well as other clade 1 sarbecoviruses [103].

In a recent study, Wang et al. engineered RBD sequences with six mutations and integrated eight RBDs from zoonotic sarbecoviruses into a single vaccine design, successfully induced cross-reactive immune responses in a murine model [81]. A trivalent RBD scaffold protein has demonstrated durable protection and cross-neutralization against JN.1 and XBB lineage [104,105]. Consequently, the conserved S2 subunit and mosaic RBD platforms represent potential antigenic targets for a next-generation universal vaccine, offering broad protection against diverse sarbecoviruses and emerging SARS-CoV-2 variants.

This body of research outlines a sophisticated, dual-pronged strategy for universal vaccine development, moving beyond the limitations of single-antigen platforms. The first approach involves targeting the highly conserved S2 subunit, essentially attacking the virus’s stable architectural core to elicit a deep and durable immune response. The second, complementary approach uses multivalent or mosaic-RBD platforms, which aims to achieve breadth by preemptively exposing the immune system to a diverse array of potential future mutational pathways. Together, these convergent strategies demonstrate that the path forward lies not in finding a single perfect epitope, but in intelligently combining conserved and variable antigens to generate a robust immune response towards the virus.

## 11. Current Universal SARS-CoV-2 Vaccine Strategies

Following the initial rollout of mRNA-based SARS-CoV-2 vaccines, research shifted toward broadly protective platforms for universal vaccine development, driven by the emergence of variants of concern (VOCs). In August 2022, the first universal SARS-CoV-2 DNA vaccine combined polyvalent RBD with the membrane and nucleoproteins, inducing cross-reactive antibodies (Wuhan-1), Beta, Delta, and Omicron variants and priming nucleoprotein-specific T cells [106]. Another multi-subunit universal vaccine provided protection against ancestral SARS-CoV-2 and Omicron subvariants (BA.1, BA.2, BA.2.75, BA.4.6, BA.5), inhibiting Omicron replication, and fully protecting mice from lethal SARS-CoV-2 Delta and SARS-CoV challenges [81]. Both vaccines show promise as universal COVID-19 and pan-sarbecovirus candidates, capable of preventing current and future SARS-CoV-2 variant and SARS-related outbreaks. An immunoinformatic-designed-epitope vaccine combining SARS-CoV-2 nucleocapsid with M2e and HA2 influenza proteins shows potential as a universal candidate [107]. Furthermore, more recently the U.S. department of Health and Human Services (HHS) announced a next-generation universal SARS-CoV-2 vaccine using a beta-propiolactone-inactivated whole-virus approach [108]. Table 2: Summary of ongoing and recent trials investigating universal vaccine platforms. Current universal vaccine efforts focus on multi-epitope designs, conserved antigens, and inclusion of antigens from other coronaviruses and sarbecoviruses to achieve broad protection against emerging variants and coronaviruses. However, this goal remains unmet to date. Indeed, the current landscape of universal vaccine development marks a clear transformation from the initial, singular focus on the spike protein. This new phase is characterized by a multi-pronged approach, experimenting with diverse platforms—from DNA to inactivated whole-virus—and combining various antigens, including conserved non-spike proteins and epitopes from multiple variants. While a definitive solution remains elusive, this collective effort underscores a consensus that achieving broad, durable protection requires abandoning simple, single-antigen designs in favor of these more complex, multi-faceted strategies.

## 12. Future Directions for SARS-CoV-2 Universal Vaccine Platforms

**Figure 3:**
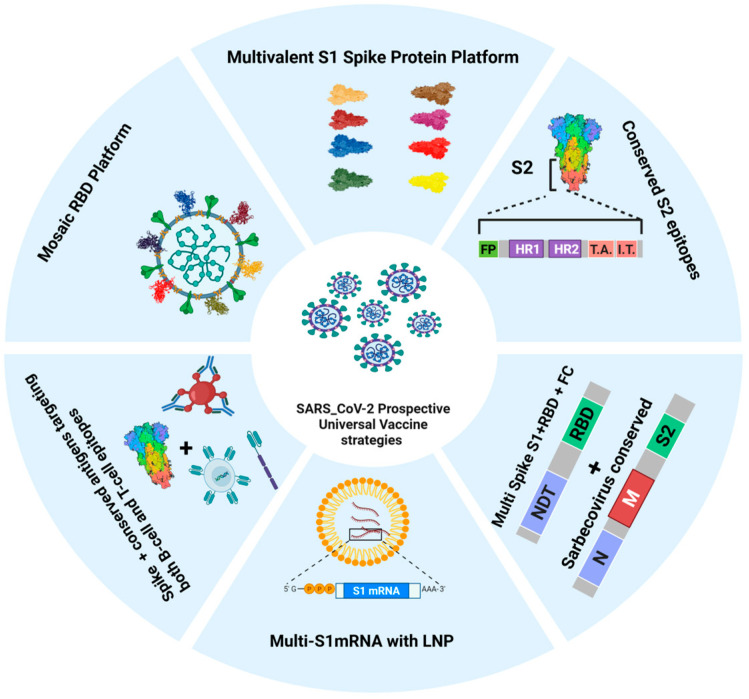
Current and Prospective Universal SARS-CoV-2 Vaccine Platforms. This schematic overview illustrates six key strategies for developing a universal coronavirus vaccine, including multivalent spike protein, mosaic RBD, multi-S1 mRNA with nanoparticles, and approaches targeting conserved S2, B-cell, and T-cell epitopes from various sarbecoviruses.

Despite the rapid deployment of COVID-19 vaccines, the ongoing viral evolution and emergence of VOCs highlight the urgent need for universal vaccine strategies capable of providing broad and durable protection. Approaches targeting conserved viral epitopes that elicit robust cell-mediated immunity and induce broadly neutralizing antibodies are essential for developing universal vaccines that offer durable protection against SARS-CoV-2 variants as well as a broad range of sarbecoviruses [85,111] and potential future zoonotic threats. The emergence of novel variants increases the need for pan-coronavirus protection, durable immune responses, and optimized antigen delivery platforms, all of which remain major challenges in next-generation COVID-19 vaccine development [112]. Such challenges necessitate utilizing new tools, including artificial intelligence (AI) and machine learning (ML) for antigen selection and vaccine design in order to facilitate the development of universal SARS-CoV-2 vaccines. These technologies can streamline antigen selection and immunogen design by predicting antigenic epitopes, assessing immunogenicity, and designing simulation models to enhance immunogen stability and coverage [113]. A notable example is Dense Convolutional Network (DenseNet), a platform developed by Chinese researchers to predict protein–ligand interactions [114], which is critical for designing a universal vaccine by assessing receptor-binding capability. Figure 3 illustrates an overview of current SARS-CoV-2 vaccine platforms and their future potential for the development of universal vaccines. Integrating one or more of these approaches will be instrumental in leveraging advanced technologies to develop novel universal SARS-CoV-2 vaccines capable of combating this highly disruptive pathogen.

## 13. Challenges in Developing and Clinically Evaluating a Universal SARS-CoV-2 Vaccine

Universal vaccines have long been envisioned as a solution to rapidly evolving viral pathogens including influenza and human immunodeficiency virus (HIV) [115]. Yet, their successful development remains constrained by multiple scientific, technological, and translational challenges. In the context of SARS-CoV-2, these challenges arise from the interplay between viral evolution, vaccine design limitations, and practical considerations related to safety, manufacturing, and implementation [116,117]. A primary scientific obstacle in universal vaccine development is the selection of an optimal vaccine target. Viral regions that remain conserved across variants serve as ideal vaccine targets because they are less likely to mutate and are therefore more likely to confer broad protection against both circulating and future variants [115,118]. However, these conserved regions are often inadequately immunogenic and rarely induce strong neutralizing antibody responses [115]. Conversely, highly immunogenic viral epitopes include those located within the spike protein receptor-binding domain (RBD) and N-terminal domain (NTD) [23]. Unfortunately, these are subject to intense immune selection pressure and consequently accumulate mutations rapidly [23,116]. Vaccines targeting these regions can provide strong protection initially but become vulnerable to immune escape as variants emerge. This trade-off between conservation and immunogenicity complicates the design of vaccines capable of inducing both potent and durable cross-variant immunity.

Ongoing viral evolution further complicates universal vaccine development. SARS-CoV-2 continues to diversify globally, generating variants with enhanced transmissibility or immune escape properties [119,120]. While advances in artificial intelligence and computational modeling allow prediction of potential mutation patterns, incorporating all predicted antigenic changes into a single vaccine is not feasible [121]. Excessively multivalent vaccine formulations would increase manufacturing complexity and regulatory challenges while also risking increased reactogenicity [122]. Highly complex antigen compositions may provoke stronger inflammatory responses or adverse reactions, potentially undermining vaccine safety and acceptance. Thus, even when future variants can be anticipated, practical immunological and safety limits restrict how much antigenic diversity can realistically be incorporated into a universal vaccine platform.

Beyond biological challenges, translational and economic considerations play a major role. Universal vaccines would require extensive preclinical studies and large-scale clinical trials to demonstrate protection across variants and populations, increasing both development time and cost [123]. Manufacturing processes must also remain adaptable to future viral evolution while maintaining scalability and affordability, particularly for global vaccination campaigns. Regulatory pathways for vaccines designed to anticipate future variants are still evolving, further complicating deployment timelines.

Collectively, these scientific and practical challenges explain the lack of a universal SARS-CoV-2 vaccine even after years of extensive funding and research. This remains true not only for SARS-CoV-2 but also for other viruses including influenza, which emerged long before and thus has certainly had significantly more funding and research. In order to pave the way for a universal vaccine, advances in antigen design and predictive modeling are crucial. Equally important are improved understanding of immune responses, scalable manufacturing strategies, and regulatory frameworks capable of supporting the development and deployment of universally protective vaccine platforms against current and future coronavirus variants.

## 14. Conclusions

Ongoing SARS-CoV-2 evolution has revealed the inherent limitations of spike-focused, variant-matched vaccines, which struggle to maintain durable and cross-variant protection. While current vaccines continue to reduce infection rates and mitigate severity of disease, a key factor driving the urgency for a universal vaccine is the significant genetic and antigenic evolution of the SARS-CoV-2 spike protein, as well as diminished protection provided by spike-based vaccines.

Universal SARS-CoV-2 vaccines offer a promising strategy to curb the emergence and re-emergence of variants by targeting conserved regions of the spike protein alone or combined with other stable elements such as S2 and leveraging multivalent platforms incorporating epitopes from related coronaviruses. These approaches aim to induce broad neutralizing antibodies and, critically, robust and durable T-cell responses. Since T-cell immunity is less affected by antigenic variation, enhancing cross-reactive cellular immunity may provide more resilient and long-lasting protection.

In summary, a universal SARS-CoV-2 vaccine platform that integrates multivalent and conserved targets with strong t-cell immunogenicity is vital for the feasibility of a universal SARS-CoV-2 vaccine for long-term preparedness against future coronavirus threats.

## Figures and Tables

**Table 1:** Neutralization reduction of available COVID-19 vaccines by variants and subvariants.

Variants/Subvariants	Neutralization	Mechanism of Escape	Type of Vaccine Antigen	Reference
Omicron BA.1, BA.1.1, and BA.2	Reduced neutralization	Escape from neutralization	mRNA vaccine	[55]
Omicron BA.1 & BA.2	Evasion from neutralization	Changing entry pathway	Viral vector, mRNA	[51]
Omicron XBB.1.5	Complete resistance to neutralization	Humoral immune escape	mRNA vaccine	[50]
Omicron XBB.1.5 & BN.1	Significantly boosted NAb levels against both ancestral and Omicron subvariants	Point mutation in spike	Bivalent mRNA Vaccine containing BA.4/5-	[56]
Omicron CH.1.1	Complete resistance to neutralization	Point mutation in spike	mRNA vaccine	[53]
Omicron EG.5 EG.5.1	1. Low neutralization	Immune evasion	Bivalent Original/Omicron BA.4/BA.5 vaccine	[52]
Omicron BQ.1.1/CH.1.1	Evasion of neutralizing antibody	Convergent mutations in RBD	inactivated virus+ homologous booster	[57]
JN.1.	Lowest neutralization across vaccines	Immune escape	Two-dose mRNA + booster with Omicron	[58]
NB1.8.1	Reduced neutralization	Substitution of Ser446Asn	Two doses of spike mRNA	[22]

**Table 2:** The current universal vaccine platforms.

Vaccine Strategies	Vaccine Platform	Induced Immunity	Vaccine Efficacy	Stage of Development	Reference
Polyvalent for multiple variants	Receptor-binding domain (RBD) for huCoV-19/WH01, Alpha, and the Beta variants	Cross-reactive spike antibodies neutralized and T-cell response	Highly cross-reactive neutralizing antibodies and T cells protective against lethal SARS-CoV-2	Pre-clinical	[106]
Subunit of conserved spike protein	Multiple variants including Omicron BA.1, Delta, and BA.5 Omicron BA.1 subvariant	Neutralizing antibodies against all SARS-CoV-2 variants tested including Alpha, Beta, Gamma, and Delta variants, the BA.1, BA.2, BA.2.75, BA.4.6, and BA.5	Complete protection against and challenge with lethal SARS-CoV-2	Pre-clinical	[81]
Live attenuated codon-deoptimized	Beta-propiolactone (BPL)-inactivated virus and codon-deoptimized	Robust antibody neuralization	Induces robust B- and T-cell immune responses and offers long-lasting protection	Pre-clinical	[75]
Multi conserved epitopes	17 conserved epitopes from S1 and S2	Broad antibody neutralization	Broadly protective immune responses, particularly CD-8^+^ T-cell response against various SARS-CoV-2 variants, and other human coronaviruses including MERS, SARS viruses	Pre-clinical	[109]
SARS-CoV-2 envelop protein expressed in Baculovirus	Recombinant SARS-CoV-2 envelop protein	Broad-spectrum protection antibody	Stimulate humoral and cellular immune responses	Pre-clinical	[110]

## Data Availability

No new data were created or analyzed in this study. Data sharing is not applicable to this article.
